# Work schedule characteristics and sleepiness – a meta-analysis

**DOI:** 10.5271/sjweh.4273

**Published:** 2026-03-01

**Authors:** Laura Vieten, Anna Arlinghaus, Marc Sobisch, Frank Brenscheidt, Dipl Wirtschaftsinformatiker, Sina Fischer, Johannes Gärtner, PD Dr

**Affiliations:** 1 Federal Institute for Occupational Safety and Health (BAuA), Dortmund, Germany.; 2 XIMES GmbH, Vienna, Austria.

**Keywords:** fatigue, night work, occupational health, shift work, work recovery, working time arrangement

## Abstract

**Objectives:**

Sleepiness is a specific aspect of fatigue and considered a key factor linking working time to health and safety outcomes, such as occupational injuries. Therefore, this meta-analysis synthesizes evidence on how specific work schedule characteristics relate to sleepiness. Specifically, we examine how sleepiness varies with: (a) shift type (eg, day versus night), (b) shift length, and (c) the number of consecutive shifts.

**Methods:**

We conducted a systematic literature search across multiple platforms and databases. Only studies that measured sleepiness using the Karolinska Sleepiness Scale were included. Mean effect sizes (Hedges' g) were calculated using random-effects models.

**Results:**

The analysis included 48 studies (28 on shift type, 30 on shift length, and 13 on shift number). Compared to day shifts, sleepiness was significantly higher during night shifts and lower during afternoon shifts. Sleepiness increased with shift length during night shifts but showed no consistent pattern during day shifts. Furthermore, sleepiness was highest on the first night shift and decreased over subsequent nights, whereas it remained relatively stable across consecutive day shifts. Due to the low number of studies, results on shift length and shift number were limited or unavailable for afternoon shifts.

**Conclusions:**

Overall, this meta-analysis shows that sleepiness is most pronounced during night shifts, particularly the first in a block. These findings emphasize the importance of circadian and homeostatic factors in shift work. Therefore, shift schedules should be designed to mitigate these heightened risks, for example by allowing sufficient recovery time before and during the first night shift.

In Europe, more than a third of employees (34%) work atypical hours (1). Given their prevalence, it is important to understand the potential consequences of such working hours. Working time arrangements have been linked to workers' performance, health, and safety (2, 3). While certain aspects, such as work-time control, have often been associated with positive outcomes (4), many characteristics of work schedules have been shown to have negative effects. Night work is well known to be associated with several adverse outcomes, including an increased risk of cardiovascular disease, gastrointestinal and metabolic disorders (5), and occupational injuries (6). Other work schedule characteristics associated with poor health and/or safety outcomes include long working hours (eg, 12-hour shifts) (6), numerous consecutive work shifts (6), and short rest periods, commonly referred to as quick returns (ie, <11 hours of rest time between shifts) (7).

Work-related fatigue is considered as a key mechanism linking different aspects of work schedules to insufficient recovery from work and more chronic health and safety outcomes (3, 8). However, fatigue is a broad and heterogeneous concept (9) that can be caused by various work-related and non-work-related factors (10). Accordingly, there is a wide range of definitions (11, 12) and operationalizations of fatigue, including physiological, behavioral, and self-report measures. To provide a more precise understanding, this study focuses specifically on sleepiness. Sleepiness can be seen as a specific form of fatigue (13), which refers to a "craving or desire for sleep" (14, p. S57). It involves physiological changes that directly affect information processing, affect, and emotion (8).

The relationship between work schedule characteristics and sleepiness can be explained by various models (eg, the Sleep/Wake Predictor, 15, 16) and frameworks (8, 13) that describe the causes of sleepiness. As many of these models and frameworks are based on Borbély's (17) two-process model of sleep regulation (3), a common assumption is that sleepiness is determined by both circadian and homeostatic factors. Circadian factors include the effects of time of day and the 'internal body clock', whereas homeostatic factors refer to the effects of time since awakening and the quantity and quality of prior sleep. Work schedule characteristics are associated with both circadian and homeostatic factors as they determine both the circadian timing of work and rest, and work duration, which affects time awake. Therefore, they can be expected to affect workers' sleepiness (8) – and, consequently, their performance, health, and safety. For instance, night shifts involve working during the period that includes the circadian nadir of alertness in the early morning, around 03:00–05:00 hours, which is why night work is expected to be related to higher levels of sleepiness than day work (8, 18). Furthermore, working a high number of consecutive night shifts compared to just a few or even a single shift might lead to increased sleepiness as the sleep deficit typically associated with night work tends to accumulate (19).

In addition to circadian and homeostatic factors, other causes of sleepiness have been discussed. For instance, Williamson et al (13) suggested that task-related factors, such as the effects of time on tasks often referred to as time on duty or 'time into shift', also play a role. In this respect, it can be argued that the work itself imposes a workload, which in turn contributes to sleepiness (18). According to these assumptions, longer daily working hours may be associated with higher levels of sleepiness than shorter ones.

In line with these theoretical assumptions, several studies have examined associations between work schedule characteristics and sleepiness. For example, a diary study of Finnish train drivers and railway traffic controllers found that the risk of severe sleepiness was particularly high during night shifts but also elevated during morning shifts compared to day shifts (20). The study also showed that the risk of severe sleepiness increased with shift length. By contrast, a Swedish study of chemical plant workers examining the effects of transitioning from an 8- to 12-hour shift system found that the 12-hour schedule was associated with lower levels of sleepiness (21). However, the authors noted that these differences might also be attributed to other schedule characteristics such as the higher number of quick returns in the 8-hour shift system.

Due to the inconsistent empirical findings and an increasing number of studies focusing on specific shift systems, occupational groups, or organizations (22), there is a need to synthesize and clarify the findings on work schedule characteristics and sleepiness. Such a synthesis would improve our understanding of the relationship between working hours and sleepiness. In addition, it would facilitate the development of evidence-based guidelines on scheduling work to mitigate the risk of excessive sleepiness. Although several reviews have addressed this topic to some extent (18, 23–26), they differ considerably in scope and focus. For instance, some examine fatigue more broadly (26), while others focus on scheduling interventions (23) or special shift systems (25). To date, no meta-analytic synthesis has comprehensively quantified the association between central work schedule characteristics and subjective sleepiness across various settings. This study aims to address this gap by examining how sleepiness changes with (a) shift type (eg, day vs. night), (b) shift length, and (c) the number of consecutive shifts. These three work schedule characteristics were chosen because they are central and well-studied aspects of working time systems that have been the focus of previous reviews (6, 26, 27).

## Methods

We conducted this study in accordance with the PRISMA 2020 Statement (28) and reported our findings accordingly. Focusing on sleepiness, it is part of a broader review project examining fatigue and the need for recovery in relation to work schedule characteristics. The broader review was pre-registered in the PROSPERO database (CRD42024537858). Any deviations from the pre-registered procedure are reported in table S1 of the supplementary material (OSF repository: https://doi.org/10.17605/OSF.IO/Z4TSU).

### Literature search

As part of the broader review project, we conducted a comprehensive literature search using the following electronic resources: PubMed (which includes records from MEDLINE, PubMed Central, and the NCBI Bookshelf), Web of Science Core Collection (via the Web of Science platform), PsycINFO, CINAHL, and PSYNDEX (all via EBSCOhost). The exact search strings are available in the supplementary material. We applied a search filter to limit the results to publications in English or German.

**Figure 1:**
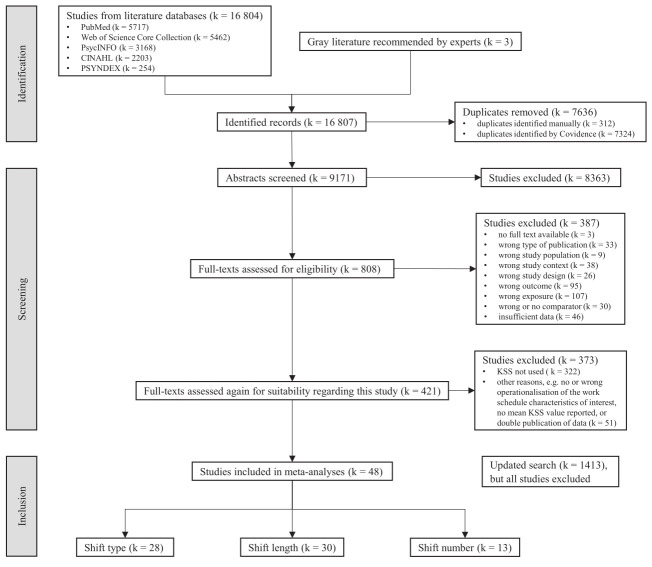
PRISMA study flow diagram.

The search was performed on 21 February 2024, and yielded 16 804 hits. To identify relevant gray literature, we consulted the members of the project's Scientific Advisory Board in June 2024 and included three additional studies based on their recommendations. After removing duplicates, 9171 studies remained. We updated the literature search on 9–10 January 2025, yielding a further 1413 hits, or 744 after removing duplicates. The PRISMA study flow diagram is shown in figure 1.

### Eligibility criteria

*Type of studies and population.* Only recommended gray literature and original, peer-reviewed journal articles published in English or German since 1970 were included. Studies were eligible if they were experimental or observational in design; descriptive, qualitative, case, and clinical trial studies were excluded. We also restricted eligibility to studies with samples including adults aged ≥16 years or workers. Studies with samples focusing on athletes, patients, animals, or special working conditions due to the COVID-19 pandemic were excluded.

*Outcome: fatigue and need for recovery including sleepiness.* Since fatigue and need for recovery can be measured by various constructs and operationalizations, we included a range of possible outcome measures in the broader review. Specifically, we were interested in studies reporting on workers' subjective need for recovery, intershift recovery, state of feeling or being recovered, mental fatigue, general physical fatigue, sleepiness, alertness, and performance on the Psychomotor Vigilance Test (PVT) or similar reaction time tests.

In this study, we focused on self-reported sleepiness as measured by the Karolinska Sleepiness Scale (KSS; 29). The KSS measures the propensity to fall asleep on a nine-point scale from ‘extremely alert' to ‘very sleepy, great effort to keep awake, fighting sleep' (18). It is a valid and reliable measure of sleepiness, with significant correlations to physiological and performance-based indicators, such as electroencephalographic activity or driving performance (18).

*Exposure: work schedule characteristics.* Studies were eligible if they reported on at least one of the following work schedule characteristics: daily or weekly work hours, shift timing, shift distribution, variability of working time, rest time between shifts, rest breaks, and work-time control. Studies were excluded if they reported only time awake without any reference to work; or used overly broad operationalizations (such as a simple comparison of shift work versus no shift work; referring to overtime without specifying working time; or referring to rest periods or shift types without specifying timing or duration).

In this study, we focused on three work schedule characteristics: shift type, shift length, and the number of consecutive shifts. *Shift type* refers to the time of day a work shift takes place. We distinguished between morning/day shifts (starting at or before 09:00 hours), afternoon/evening shifts, and night shifts (≥6 hours of work between 22:00–06:00, according to the Austrian Heavy Night Work Act). *Shift length* refers to the time elapsed since the start of a work shift. We included studies that reported sleepiness levels at multiple time points during a shift, for example, at the beginning and end or at hourly intervals. Time into shift was classified on an hourly basis from 1–>12 hours, with 1 referring to the time from the start of the shift until the end of the first work hour. *Shift number* refers to the number of consecutive shifts worked. For day shifts, only blocks surrounded by days off were included. For night shifts, blocks were fully surrounded by days off in five studies. In four studies, night shift blocks were preceded by other shift types and, in one study, a block was followed by two afternoon shifts.

*Further eligibility criteria.* We included only studies that compared relevant groups or levels. For example, studies comparing sleepiness among night shift and day shift workers were excluded if these groups belonged to different occupational categories. Furthermore, studies had to report statistical effect sizes for the exposure-outcome associations of interest or provide information to calculate them.

### Screening process

We used Covidence software (www.covidence.org) for screening. Five authors independently screened titles and abstracts, with two reviewers assessing each record. Full texts were also screened in pairs. Inter-rater agreement ranged from moderate to almost perfect (abstracts: 80–99%, full texts: 71–86%). Disagreements were resolved either by a third reviewer or consensus within the review team. We excluded 8363 studies after title and abstract screening and 387 studies after full-text assessment, leaving 421 studies (see figure 1).

The full texts of these 421 studies were re-screened for quality and relevance to this study's specific research question. We included only studies that reported mean KSS values (or sufficient data to calculate them) for at least two distinct and comparable categories of the work schedule characteristics of interest, including a reference category. In total, 48 studies were considered eligible (see figure 1).

A single reviewer screened records from the updated search with a focus on this study's research question. Uncertainties were discussed within the review team. However, no further studies met the inclusion criteria.

### Data extraction

The author team extracted data for the meta-analysis. Key study characteristics were coded, including study design, location, sample details (eg, size, mean age, percentage of women, working time system), as well as the type and timing of exposure and outcome measures and their associations. In many studies, KSS values were not reported as exact numerical values, but presented graphically. In such cases, we extracted numerical estimates (eg, means and standard errors or standard deviations) from figures using WebPlotDigitizer software (apps.automeris.io/wpd4). If only mean values were reported, the standard deviation was imputed based on the other studies for each work schedule characteristic. For instance, regarding shift type, we calculated the average standard deviation separately for day, afternoon and night shifts. After coding, a second coder checked all data to ensure accuracy. Discrepancies or uncertainties were resolved through team discussion.

### Statistical analyses

We estimated mean differences for repeated measures using random-effects models for all work schedule characteristics if at least three studies reported values for the respective comparison. Hedges' g was calculated as the effect size, along with its 95% confidence interval (CI), 95% prediction interval (PI), and two-tailed P-value. Effect sizes and meta-analyses were computed using Meta-Essentials 1.5 (30), with CI calculated with the weighted variance method (31).

For shift type, we calculated the mean difference between day and night shifts and between day and afternoon shifts. As most studies used repeated-measurement designs, we calculated the mean differences for dependent samples. To account for correlation between measurements (32) in Meta-Essentials, we derived correlations from a single study (33) that reported t-statistics: day–night r=0.47, day–afternoon r=0.56. Studies using between-subjects designs were included with r=0.00.

For shift length, we used the first hour of the shift as the reference and calculated mean differences for each subsequent hour. Analyses were conducted separately for day, afternoon, and night shifts. As no study reported the correlation between time points, we assumed r=0.50.

For shift number, we used the first shift as a reference and calculated mean differences for each subsequent shift. Analyses were limited to day and night shifts because too few studies reported on consecutive afternoon shifts. Again, we assumed a correlation of r=0.50.

Heterogeneity was assessed using Cochran's Q test and the I^2^ statistic. If the Q test was significant, moderator and subgroup analyses were performed. However, these analyses were only performed for shift type due to the small number of studies for some categories of shift length and number (<10 studies; 34). The moderators included mean age, proportion of women, shift length, and start time of day shifts. For the subgroup analyses, the studies were grouped by sector (healthcare, industry, and other) and by study setting (field versus laboratory).

Sensitivity analyses were conducted to assess the robustness of the results. In one iteration, the correlation between measurements was set to r=0.00. In subsequent iterations, studies were excluded based on specific criteria, including extreme effect sizes (largest and smallest), highest weight, a sample of offshore workers, and day shifts starting before 06:00 hours.

We also assessed potential publication bias using Egger's regression test for funnel plot asymmetry (35), visual inspection of funnel plots, and trim-and-fill method (36). These methods were only applied to analyses based on ≥10 studies (37). The results are reported in the supplementary material.

## Results

### Study characteristics

We included 48 studies in our meta-analysis. Of these, 28 reported KSS values for shift type, 30 for shift length, and 13 for shift number. Twenty-two of these studies contributed data to more than one work schedule characteristic. The studies were published between 1994 and 2022 and came from 13 different countries, most frequently Sweden (k=11), followed by the USA (k=8). While the original studies often included larger overall samples, the average analyzed sample size was 6–144 participants. Most studies examined nurses (k=13), though other occupational groups, such as drivers, offshore workers, or physicians, were also represented. Further information on study design and participant characteristics (mean age and percentage of women) is presented in table 1.

**Table 1:** Description of study characteristics. IDs are unique identifiers generated by Covidence. [W-S=within subjects, B-S=between subject; F=field; L=laboratory; HC=healthcare; T=type; L=length; N=number; N/A=not available.]

ID	Study	Country	Study design (setting)	N	Occupation (sector)	Age: Mean (± SD or range)	Gender: % female	Exposure
14082	Åhsberg et al (55), 2000	Sweden	W-S (F)	75	Paper mill workers (other)	41.3 (10.0)	48 ^a^	T
4984	Anderson et al (56), 2018	USA	W-S (F)	16	Medical residents (HC)	29.2 (2.0)	63	L
14275	Axelsson et al (57), 1998	Sweden	W-S (F)	31	Power plant workers (other)	36.8 (11.1)	13	T, L
8814	Bjorvatn et al (40), 1998	Norway	W-S (F)	6	Oil platform workers (other)	38.9 (29–47) ^b^	0 ^b^	L, N
2935	Bonnefond et al (58), 2006	France	W-S (F)	Young: 13	Aircraft technical maintenance staff (industry)	31.0 (25–34)	0	T
				Middle: 17		45.0 (35–49)		
				Senior: 19		53.0 (50–58)		
5452	Costa et al (59), 2014	Italy	W-S (F)	A: 10; B: 10: C: 10	Nurses (HC)	34.3 (23–46)	57	T, L
5485	Dahlgren et al (60), 2006	Sweden	W-S (F)	15	Office workers (other)	45.9 (15.0)	60	N
5481	Da Silva Borges & Fischer (61), 2003	Brazil	W-S (F)	20	Nurses (HC)	34.9 (7.5)	85	L
9569	Di Muzio et al (33, 2019)	Italy	W-S (F)	14	Nurses (HC)	36.8 (8.9)	71	T
3198	Di Muzio et al (62), 2021	Italy	W-S (F)	Forward rotating: 80	Nurses (HC)	40.4 (8.9)	63	T
				Backward rotating: 64		42.3 (10.4)	66	
3301	Ferreira et al (63), 2017	Brazil	B-S, W-S (F)	Day: 36	Nurses (HC)	30.0 (N/A)	97	T, L
				Night: 32				
10012	Ganesan et al (45), 2019	Australia	W-S (F)	35	Nurses and doctors (HC)	33.8 (9.7) ^c^	72 ^c^	T, N
5843	Ganesan et al (64, 2022)	Australia	W-S (F)	First night: 13	Mining haul truck drivers (other)	34.2 (10.0)	15	L
				Second night: 16		32.7 (10.4)	38	
5871	Geiger-Brown et al (38), 2012	USA	B-S, W-S (F)	Day: 39	Nurses (HC)	37.2 (10.4)	100	T, L
				Night: 41				
10059	Geiger-Brown et al (39), 2014	USA	W-S (F)	40 ^d^	Nurses (HC)	30.9 (7.9)	N/A	T, L
15721	Gillberg (65), 1998	Sweden	B-S, W-S (F)	Day: 9	Production workers (industry)	32.9 (6.6) ^e^	33 ^e^	T, L
				Night: 17		36.5 (8.5) ^e^		
15723	Gillberg et al (66), 2003	Sweden	W-S (L)	12	Control room operators (other)	41.0 (32–54)	0	T, L
6002	Hakola et al (67), 1996	Finland	W-S (L)	Men: 9	Postal workers (other)	40.6 (13.2)	0	T, L, N
				Women: 11		38.8 (16.6)	100	
10302	Hakola et al (68), 2021	Finland	W-S (F)	A: 10	Aircraft inspectors (other)	50.7 (4.1)	13	T
				B: 13		43.5 (6.3)		
10354	Härmä et al (69), 1994	Finland	W-S (L)	Younger: 7	Letter sorters (other)	23.7 (4.2)	57	L, N
				Older: 7		56.7 (2.0)	57	
6026	Härmä et al (70), 2006	Finland	W-S (F)	Younger: 28 ^f^	Aircraft technical maintenance staff (industry)	36.0 (30–43) ^g^	0	T
				Older: 21 ^f^		50.0 (45–61) ^g^		
10368	Harrison et al (71), 2020	USA	W-S (F)	Experiment 1: 31	Emergency medicine residents (HC)	30.8 (2.4)	42	T
				Experiment 2: 21	Emergency medicine residents and physicians (HC)	35.7 (3.3–5.1) ^h^	43	T, L
10598	Husby et al (72), 2014	Norway	W-S (F)	18	Anaesthesiology residents (HC)	35.0 (31–48)	28	L
6197	Ingre et al (73), 2004	Sweden	W-S (F)	17	Train drivers (other)	50.0 (44–60)	0	L
10647	Isherwood et al (74), 2020	USA	W-S (L)	9	Non-shift workers (other)	57.9 (4.6) ^i^	33	L
3615	James et al (75), 2021	USA	B-s (L)	94 ^j^ (Day: N=44, Night: N=49)	Nurses (HC)	35.9 (9.5)	89	T
3686	Kazemi et al (76), 2016	Iran	W-S (F)	60	Petrochemical control room operators (other)	30.1 (2.5)	0	L
3688	Kazemi et al (77), 2018	Iran	W-S (F)	Fourth night: 40	Petrochemical firefighters (other)	29.2 (1.9)	N/A	L
				Seventh night: 40		31.1 (2.6)		
6329	Kecklund et al (78), 1997	Sweden	B-S (F)	22 (Early: N=12, Control: N=10)	Airline cabin crew members (other)	37.3 (1.8–2.4) ^k^	100	T
10874	Kecklund et al (79), 2001	Sweden	W-S, F	48	Construction workers (industry)	41.0 (22–62)	0	L
10911	Khan et al (80), 2021	Australia	W-S, F	12	Paramedics (HC)	39.5 (10.7)	58	T, L
3788	Lancman (81), 2016	Australia	W-S, F	10	Anaesthesia trainees (HC)	N/A	N/A	L, N
3813	Legault et al (82), 2017	Canada	W-S, F	14	Underground development miners (industry)	41.5 (5.1) ^l^	0	T
11372	Lowden et al (21), 1998	Sweden	W-S, F	14	Control room operators (other)	37.0 (1.7) ^m^	12 ^m^	L, N
6835	Mulhall et al (83), 2019	Australia	W-S, F	33 ^n^	Nurses (HC)	34.1 (11.4)	79	T, L
6856	Narciso et al (84), 2016	Brazil	W-S, F	20	Polysomnography technicians (industry)	35.1 (7.0)	75	L
11969	Nordin & Knutsson (85), 2001	Sweden	W-S, F	16	Paper mill workers (other)	44.0 (9.7)	0	N
4131	Onninen et al (86), 2020	Finland	W-S (F)	23	Tram drivers (other)	40.6 (11.4) ^o^	48	T, L
7041	Persson et al (87), 2003	Sweden	W-S (F)	Intervention: 38	Construction workers (industry)	39.2 (10.4) ^p^	0	N
				Control: 23		42.5 (13.9)		
4278	Reinke et al (88), 2015	Netherlands	W-S (F)	42	Nurses (HC)	41.9 (2.1–10.6) ^q^	74 ^q^	T
4293	Riethmeister et al (89), 2018	Netherlands	W-S (F)	42	Offshore workers (other)	42.0 (12.1)	0	L, N
7295	Sallinen et al (90), 2020	Finland	W-S (F)	22	Long-haul truck drivers (other)	39.5 (9.5)	0	L
12918	Shochat et al (91), 2019	Israel	W-S (F)	39	Airline ground crew managers (other)	38.9 (8.2)	49	T
4692	Van Dongen et al (92), 2011	USA	B-S, W-S (L)	Day: 14	N/A (other)	27.5 (5.6)	50	T, N
				Night: 13		27.0 (5.4)	54	
13540	Vangelova (93), 2008	Bulgaria	W-S (F)	Forward rotating: 13	Sound engineers (industry)	45.1 (7.3)	69	T, L
				Backward rotating: 12		51.7 (6.0)	67	
4733	Waage et al (94), 2012	Norway	W-S (F)	15	Oil rig workers (other)	44.0 (28–60) ^r^	32 ^r^	T, N
7929	Wilson et al (95), 2019	USA	B-S, W-S (F)	Day: 11; Night: 11	Nurses (HC)	N/A (20–60)	91	T, L
4881	Zion et al (96), 2018	Israel	W-S (F)	92	Nurses (HC)	39.3 (9.1) ^s^	100	L

^a^ Value refers to a larger sample (N=92); ^b^ Value refers to a larger sample (N=7); ^c^ Value refers to a larger sample (N=50); ^d^ In the analyses for 'number": N=34; ^e^ Value refers to a larger sample (Day: N=10, Night: N=20, Total: N=30); ^f^ The paper itself does not specify an exact number. Therefore, we have calculated the number based on the following information: The study included 64 younger and 49 older workers from the old shift system. Of these, 49 participated in the field survey; ^g^ Value refers to a larger sample (Young: N=64, Older: N=49); ^h^ Mean age was calculated as a weighted average of the two subgroups of emergency medicine residents and physicians; ^i^ Value refers to a larger sample (N=27); ^j^ One nurse was measured after both a night shift and a day shift; ^k^ Mean age was calculated as a weighted average of the two subgroups, 'early" and 'control"; ^l^ Value refers to a larger sample (N=19); ^m^ Value refers to a larger sample (N=34); ^n^ In the analyses for 'length": N=28 (night), N=30 (day), N=27 (afternoon); ^o^ Value refers to a larger sample (N=158); ^p^ Value refers to a larger sample (N=41); ^q^ Value refers to a larger sample (N=96). Mean age was calculated as a weighted average of the two subgroups, 'evening" and 'morning" chronotype; ^r^ Value refers to a larger sample (N=19); ^s^ Value refers to a larger sample (N=109).

### Meta-analytic results

**Figure 2:**
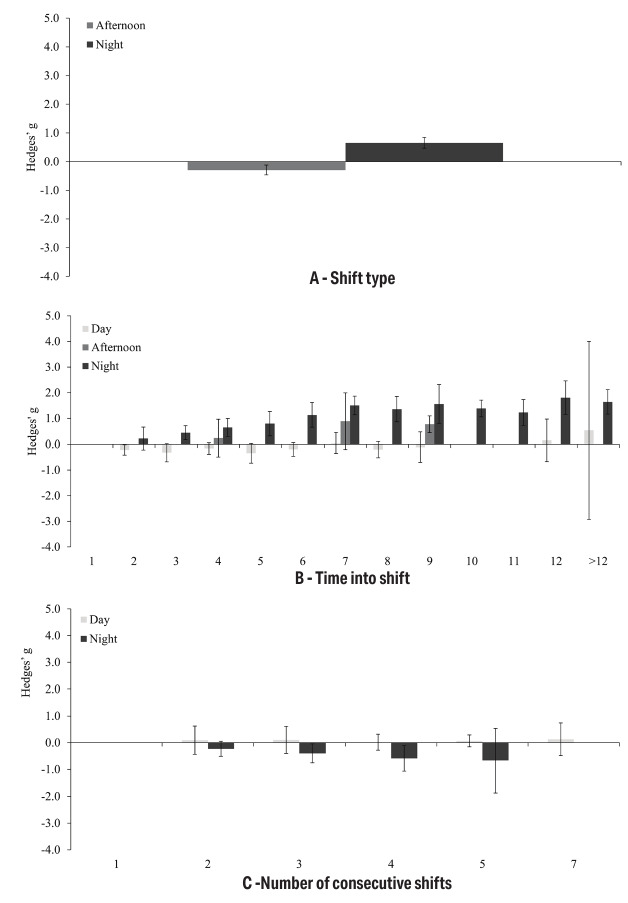
Meta-analytic result for shift type (a), shift length (b), and shift number (c). Hedges' g for sleepiness. Error bars represent 95% confidence intervals. Heterogeneity (I^2^) values are reported in the Results section. Reference categories are day shift (shift type), first hour (shift length), and first shift (shift number).

Mean KSS values extracted from the individual studies are summarized in tables 2–4. The main results are presented below and in figure 2; all additional results and analysis files are available in the supplementary material.

**Table 2:** Extracted KSS scores for shift type.

ID and study	Shift type		
	Day	Night	Afternoon
14082 Åhsberg et al (2000)	4.59	6.64	4.53
14275 Axelsson et al (1998)	4.10	5.10	
2935 Bonnefond et al (2006): Young	4.81	6.00	3.75
2935 Bonnefond et al (2006): Middle	4.16	5.41	3.75
2935 Bonnefond et al (2006): Senior	3.96	5.57	3.89
5452 Costa et al (2014): A	2.55	3.57	
5452 Costa et al (2014): B	3.61	5.20	3.14
5452 Costa et al (2014): C	3.20	4.86	2.52
9569 Di Muzio et al (2019)	3.11	4.80	3.56
3198 Di Muzio et al (2021): Forward rotating	5.37	6.90	5.08
3198 Di Muzio et al (2021): Backward rotating	7.01	8.97	6.89
3301 Ferreira et al (2017)	4.14	3.33	
10012 Ganesan et al (2019)	4.45	5.21	
5871 Geiger-Brown et al (2012)	3.10	3.23	
10059 Geiger-Brown et al (2014)	4.10	4.90	
15721 Gillberg (1998)	5.88	4.84	
15723 Gillberg et al (2003)	4.87	5.14	
6002 Hakola et al (1996): Men	2.96	4.79	
6002 Hakola et al (1996): Women	3.24	3.87	
10302 Hakola et al (2021): A	4.38	6.24	3.34
10302 Hakola et al (2021): B	5.68	5.72	3.40
6026 Härmä et al (2006): Younger	4.58	6.10	
6026 Härmä et al (2006): Older	4.13	5.44	
10368 Harrison et al (2020): Experiment 1	4.10	5.12	3.39
10368 Harrison et al (2020): Experiment 2	3.67	4.95	4.01
3615 James et al (2021)	4.65	5.12	
6329 Kecklund et al (1997)			4.54
10911 Khan et al (2021)	4.84	6.03	
3813 Legault et al (2017)	4.43	4.38	
6835 Mulhall et al (2019)	4.49	5.38	3.47
4131 Onninen et al (2020)			3.66
4278 Reinke et al (2015)	3.45	4.86	
12918 Shochat et al (2019)	3.02	3.12	
4692 Van Dongen et al (2011)	2.76	3.98	
13540 Vangelova (2008): Forward rotating	5.63	7.15	4.40
13540 Vangelova (2008): Backward rotating	5.74	7.53	4.19
4733 Waage et al (2012)	3.40	3.34	
7929 Wilson et al (2019)	4.12	4.72	
Unweighted mean: Day vs. night	4.23	5.21	
Unweighted mean: Day vs. afternoon	4.62		3.97

**Table 3:** Extracted KSS scores for shift length.

ID and study		Time (hour)												
		1	2	3	4	5	6	7	8	9	10	11	12	>12
**Time into a night shift**														
	14275 Axelsson et al (1998)	3.82		4.69		5.84		6.11						
	8814 Bjorvatn et al (1998)	4.36		4.46		4.74		5.28		5.71		6.10		
	5452 Costa et al (2014): A	1.47					3.30						5.93	
	5452 Costa et al (2014): B	4.26				5.78					5.56			
	5452 Costa et al (2014): C	3.49				5.17					5.93			
	5481 Da Silva Borges & Fischer (2003)	3.00			4.30			5.30			6.30			
	3301 Ferreira et al (2017)	2.24											4.41	
	5843 Ganesan et al (2022): First night	4.08									6.25			
	5843 Ganesan et al (2022): Second night	4.06									6.62			
	5871 Geiger-Brown et al (2012)	2.46	2.29		2.57		3.32		4.01		4.07		3.87	
	15721 Gillberg (1998)	4.10		4.22				5.03					6.00	
	15723 Gillberg et al (2003)	3.40	3.81	4.50	5.21	5.50	5.79	6.41	6.50					
	6002 Hakola et al (1996): Men	4.02	4.68	4.36	4.77	4.95	5.54	6.02						
	6002 Hakola et al (1996): Women	3.01	3.60	3.52	3.57	3.78	4.75	5.69						
	10354 Härmä et al (1994): Younger	2.76		3.55	3.90	4.07	4.58	5.62						
	10354 Härmä et al (1994): Older	2.91		3.49	3.47	3.50	4.49	4.97						
	10368 Harrison et al (2020): Experiment 2	4.00			4.55				6.29					
	10589 Husby et al (2014): 10h	3.93									6.33			
	10589 Husby et al (2014): 18h	3.54												6.54
	10647 Isherwood et al (2020)	3.31	4.01	4.69	5.08	4.94	6.08	6.39	6.57					
	3686 Kazemi et al (2016)	1.67		1.88		2.19		3.36		4.46			5.15	5.05
	3688 Kazemi et al (2018): Fourth night	2.48				1.23				6.63				5.60
	3688 Kazemi et al (2018): Seventh night	2.86				3.13				4.30				6.50
	10911 Khan et al (2021)	4.67												7.70
	3788 Lancman (2016)	3.64										6.21		
	11372 Lowden et al (1998)	4.64				5.99				7.14				
	6835 Mulhall et al (2019)	4.08										6.68		
	6856 Narciso et al (2016)	3.45												5.50
	7295 Sallinen et al (2020)	3.24					4.45					5.00		
	13540 Vangelova (2008): Forward rotating	5.74			6.83			8.89						
	13540 Vangelova (2008): Backward rotating	6.06			7.40			9.13						
	7929 Wilson et al (2019)	3.90					4.38						5.89	
	4881 Zion et al (2018)	2.69	2.52	2.76	3.21	3.67	4.59	5.20	4.67	4.97				
	Unweighted mean for night shifts	3.56	3.48	3.83	4.57	4.30	4.66	5.96	5.61	5.53	5.87	6.00	5.21	6.15
**Time into a day shift**														
	4984 Anderson et al (2018)	3.98								4.03				
	14275 Axelsson et al (1998)	4.67		3.77		3.79		4.11						
	5452 Costa et al (2014): A	1.70					2.06						3.90	
	5452 Costa et al (2014): B	2.89			3.22			4.73						
	5452 Costa et al (2014): C	3.10			2.98			3.52						
	3301 Ferreira et al (2017)	4.17											4.11	
	5871 Geiger-Brown et al (2012)	3.06	2.53		2.66		2.84		3.06				3.95	
	15721 Gillberg (1998)	6.26			5.92				5.58				5.75	
	15723 Gillberg et al (2003)	5.19	5.00	4.79	5.31	4.80	4.70	4.70	4.50					
	6002 Hakola et al (1996): Men	3.23	2.88	2.55	2.66	2.67	3.12	3.32						
	6002 Hakola et al (1996): Women	3.45	3.45	3.37	3.10	3.07	2.99	3.02						
	10368 Harrison et al (2020): Experiment 2	4.32			2.68				4.00					
	10589 Husby et al (2014)	3.21												6.07
	6197 Ingre et al (2004)	3.37	3.24	3.47	3.58	3.77								
	3686 Kazemi et al (2016)	3.07		2.57		2.34		2.54		3.00			1.95	2.09
	10874 Kecklund et al (2001)	3.65			3.49				3.51					
	6835 Mulhall et al (2019)	5.13								3.85				
	4131 Onninen et al (2020)	3.85								4.11				
	4293 Riethmeister et al (2018)	4.02												4.59
	13540 Vangelova (2008): Forward rotating	5.26			5.31			6.32						
	13540 Vangelova (2008): Backward rotating	5.51			5.71			6.00						
	7929 Wilson et al (2019)	4.22					3.55						4.60	
	Unweighted mean for day shifts	3.97	3.42	3.42	3.88	3.41	3.21	4.25	4.13	3.75			4.04	4.25
**Time into an afternoon shift**														
	5452 Costa et al (2014): B	3.42			2.42			3.60						
	5452 Costa et al (2014): C	1.98			2.23			3.36						
	10368 Harrison et al (2020): Experiment 2	3.29			3.71									
	11372 Lowden et al (1998)	3.07								4.31				
	6835 Mulhall et al (2019)	2.83								4.10				
	4131 Onninen et al (2020)	2.84								4.47				
	13540 Vangelova (2008): Forward rotating	3.51			3.92			5.77						
	13540 Vangelova (2008): Backward rotating	2.63			4.48			5.45						
	Unweighted mean for afternoon shifts	2.95			3.35			4.54		4.29				

**Table 4:** Extracted KSS scores for shift number.

ID and study		Number of consecutive shifts						
		1	2	3	4	5	6	7
**Night shifts**								
	8814 Bjorvatn et al (1998)	6.79	5.26	4.79	4.84	4.71		
	10012 Ganesan et al (2019)	5.62			4.79			
	10059 Geiger-Brown et al (2014)	4.70	5.00	5.10				
	6002 Hakola et al (1996): Men	4.83	5.03	4.53				
	6002 Hakola et al (1996): Women	4.79	3.72	3.09				
	10354 Härmä et al (1994): Younger	4.99	4.03	2.72				
	10354 Härmä et al (1994): Older	3.67	3.93	3.50				
	3788 Lancman (2016)	5.27	4.96	4.52	4.68	5.13		
	11372 Lowden et al (1998)	6.44	6.03	5.80	5.42			
	11969 Nordin & Knutsson (2001)	5.43		5.46				
	4692 Van Dongen et al (2011)	4.72	4.13	3.80	3.55	3.71		
	4733 Waage et al (2012)	4.02	3.73	3.55	3.37	3.35		
	Unweighted mean for night shifts	5.11	4.58	4.26	4.44	4.23		
**Day shifts**								
	5485 Dahlgren et al (2006)	3.68	4.21	4.21	3.91	3.40		
	7041 Persson et al (2003): Intervention	2.05				2.21		2.24
	7041 Persson et al (2003): Control	2.30				2.52		
	4293 Riethmeister et al (2003)	3.99	4.05	4.15	4.06	4.19		4.43
	4692 Van Dongen et al (2011)	2.80	2.67	2.70	2.66	2.95		
	4733 Waage et al (2012)	3.54	3.40	3.36	3.29	3.35		3.23
	Unweighted mean for day shifts	3.06	3.58	3.60	3.48	3.10		3.30

### Shift type

There was a significantly higher level of sleepiness during the night shift compared to the day shift, reflected in a moderate positive effect size (g*=*0.66, CI 0.47–0.84, PI −0.26–1.57, k*=*36, P<0.001). Heterogeneity was high and significant (I^2^=77%, P<0.001), indicating substantial variability in the studies included, which was also underlined by PIs crossing zero. In contrast, a small but also significant negative effect was found for the afternoon shift relative to the day shift (g*=*−0.29, CI −0.46–−0.12, PI −0.80–0.22, k*=*18, P=0.002), indicating lower levels of sleepiness during the afternoon shift. Heterogeneity was substantial and significant (I^2^=59%, P<0.001). Sensitivity analyses did not substantially change these results, neither for night nor for afternoon shifts. Specifically, the respective effects remained statistically significant across all iterations, indicating that the findings are robust.

### Shift type: moderation and subgroup analysis

Due to the substantial or even high amount of heterogeneity, we tested potential moderating effects. For night shifts, only shift length showed a significant effect on the effect sizes found (b*=*−0.13, SE*=*0.04, Z*=*−3.02, P=0.003, R^2^=0.19), indicating that as shift length increased, the observed difference in sleepiness between night and day shifts decreased. For afternoon shifts, we found significant moderating effects of shift length (b=−0.21, SE=0.09, Z=−2.34, P=0.019, R^2^=0.24), and of the start time of day shifts (b=0.28, SE=0.12, Z=2.35, P=0.019, R^2^=0.27). This indicates that, as shift length increased, the observed difference in sleepiness between afternoon and day shifts increased, while a later start of the day shift was associated with a decreased difference between the two types of shifts.

### Shift length

Compared to the 1^st^ hour of a night shift, the level of sleepiness increased steadily from the 2^nd^ to the 7^th^ hour of work (2^nd^ hour: g=0.22, CI −0.22–0.66, PI −0.64–1.09, k=6, P=0.252; 7^th^ hour: g=1.50, CI 1.14–1.87, PI 0.61–2.39, k=14, P<0.001). After the 7^th^ hour, effect sizes fluctuated at a high level (g=1.23–1.80), reflecting large effects in terms of increased sleepiness compared to the 1^st^ hour. Except for the 2^nd^ hour, all effects were statistically significant. Heterogeneity varied considerably across the analyses, ranging from very low to high values (I^2^=0–88%), and was statistically significant for all analyses except for the 8^th^, 10^th^, 11^th^, and >12^th^ hour. Sensitivity analyses did not lead to any change in the (non)significance of the effect sizes, indicating that these findings are robust.

In contrast, no clear trend was observed over the day shift. Both negative and positive small-to-moderate effect sizes were found (g=−0.35–0.54), ie, sleepiness levels increased but also decreased over time into shift compared to the 1^st^ hour of work. However, only the difference between the 2^nd^ and 1^st^ hour reached statistical significance (g=−0.22, CI −0.42–−0.02, PI −0.42–−0.02, k=5, P=0.039).

Heterogeneity ranged from very low to very high values (I^2^=0–96%) and was statistically significant for all analyses except for the 2^nd^, 6^th^, and 8^th^ hour. Sensitivity analyses showed that the difference between the 1^st^ and 2^nd^ hour was not significant when the study with the highest weight (38) was excluded (g=−0.11, CI −0.24–0.02, k=4, P=0.071). When the studies with the highest and lowest effect sizes were excluded, the results became significant for the difference between the 1^st^ and the 3^rd^ (g=−0.28, CI −0.48–−0.08, k=4, P=0.020), 5^th^ (g=−0.39, CI −0.55–−0.24, k=4, P=0.004), and 6^th^ hours (g=−0.20, CI −0.36–−0.04, k=4, P=0.030).

Due to the small number of studies found, analyses could only be calculated for the 4^th^, 7^th^, and 9^th^ hours compared to the 1^st^ hour of an afternoon shift. The three effects sizes were all positive and small-to-large (g=0.23–0.89), indicating higher levels of sleepiness on the respective hour compared to the 1^st^ hour of work. However, only the difference between the 9^th^ and 1^st^ hour were statistically significant (g=0.77, CI 0.45–1.09, PI 0.45–1.09, k=3, P=0.009). Heterogeneity was low-to-substantial (I^2^=0–75%) and significant for the 4^th^ (I^2^=70%, P=0.009) and 7^th^ hour (I^2^=75%, P=0.008). Sensitivity analyses did not lead to any changes.

### Shift number

The level of sleepiness decreased with each subsequent night shift compared to the 1^st^ night (2^nd^ night: g=−0.23, CI −0.51–0.05, PI −0.85–0.39, k=10, P=0.100; 5^th^ night: g=−0.67, CI −1.87–0.53, PI −2.63–1.30, k=4, P=0.175). However, despite this systematic trend, the results were significant only for the 3^rd^ and 4^th^ night shifts (3^rd^ night: g=−0.40, CI −0.76–−0.04, PI −1.31–0.51, k=11, P=0.031; 4^th^ night: g=−0.59, CI −1.07–−0.11, PI −1.42–0.25, k=6, P=0.025). Heterogeneity was moderate-to-substantial (I^2^=44–69%) and significant for the 3^rd^ (I^2^=64%, P=0.002) and 5^th^ night shift (I^2^=69%, P=0.023). Substantial variability in the studies was also underlined by the fact that all PI included zero.

Regarding sensitivity analyses, the difference between the 1^st^ and 2^nd^ night turned significant, if the studies with highest (39) and lowest (40) effect size were excluded (g=−0.24, CI −0.46–−0.02, k=8, P=0.035) and if the study with the highest weight (39) was excluded (g=−0.30, CI −0.59–−0.00, k=9, P=0.047). The difference between the 1^st^ and 3^rd^ night turned out non-significant, if the correlation between measurements was set to r=0.00 (g=−0.33, CI −0.65–0.00, k=11, P=0.050). Further sensitivity analysis did not result in any substantial changes.

Compared to the 1^st^ shift, sleepiness levels did not change significantly during the subsequent day shifts (2^nd^–5^th^ and 7^th^) with effect sizes of g*=*0.01–0.13 (all CI and PI included zero and all P-values were not significant). Heterogeneity was low-to-moderate (I^2^=0–49%) and not significant. However, given the small number of studies included (k=3–6), these assessments should be interpreted with caution. Sensitivity analysis did not result in any substantial changes, ie, all effect sizes remained not significant.

## Discussion

Sleepiness is a specific aspect of fatigue and considered a central mechanism linking working time arrangements to workers' health and safety outcomes (3, 8). To better understand these relationships, this meta-analysis synthesized findings from 48 studies on three specific work schedule characteristics and sleepiness measured via the KSS. The theoretical implications and explanatory mechanisms of the main results are discussed below.

Regarding shift type, sleepiness was significantly higher during night shifts and significantly lower during afternoon shifts than during day shifts. These results align with theoretical assumptions describing how circadian factors influence sleepiness throughout the day (15–17). Homeostatic factors may also contribute: Night work requires sleep during daytime hours, which is typically shorter (41) and less restorative, potentially increasing sleepiness.

Regarding shift length, sleepiness steadily increased during night shifts until the 7^th^ hour, after which it remained high. For afternoon shifts, limited data also suggested an increase over time. However, no consistent pattern emerged for day shifts. These findings indicate that sleepiness may increase over the course of a shift, particularly during night shifts. However, the absence of a consistent effect across all shift types challenges the assumption that time-into-shift or time-on-duty has a consistent or sufficiently strong effect on sleepiness. This reflects the ongoing debate as to whether sleepiness, unlike other forms of fatigue, is directly caused by time spent working (18), or not (8). Our results cannot resolve this debate, but they suggest that any potential effect of time on duty is difficult to disentangle from circadian and homeostatic influences. In other words, sleepiness is shaped by the relative contributions of multiple influences, including circadian, homeostatic, and potentially time-on-duty processes, whose strength may vary across the shift and sometimes overshadow one another. For example, the increase in sleepiness during night shifts aligns with the circadian trough in the early morning (8, 18). In contrast, during day shifts, the circadian-driven reduction in sleepiness from morning to daytime hours (42), and in some cases, the gradual dissipation of sleep inertia – particularly in professions with short or no commutes, such as truck drivers or oil rig workers – could counteract any time-on-duty effect.

Furthermore, these inconsistent findings may be due to other factors. For example, work breaks could counteract sleepiness. Although we assume that most shifts under study did include some rest breaks, we often lacked specific information about break times, and therefore could not separate this effect. Moreover, workers may allocate their resources based on their shift length, adjusting their work (performance) accordingly to avoid excessive sleepiness. Alternatively, they may increase their work effort to compensate for increased sleepiness, which could be facilitated by the stimulating effect of work activity itself (43, 44).

Regarding shift number, sleepiness was highest during the first night shift and decreased over subsequent nights, while it remained relatively stable across consecutive day shifts. Contrary to expectations, our findings suggest that sleepiness does not systematically accumulate across consecutive shifts, whether day or night. Notably, some studies have found an association between consecutive night shifts and an accumulated sleep debt (eg 19,). One possible explanation for our finding is that a substantial number of workers often get sufficient rest between shifts, meaning they start each shift well-rested. However, as our analyses did not account for rest periods, this assumption cannot be verified. Therefore, future meta-analyses should investigate the influence of the duration and quality of rest periods, including sleep, on the relationship between shift number and sleepiness.

Interestingly, sleepiness did not increase from one night shift to the next; in fact, it decreased. A closer look at the average sleepiness scores across consecutive shifts (see table 4) suggests that the observed decrease was primarily due to a first-night effect. Sleepiness is particularly high during the first night shift, rather than being low from the second night shift onwards. One possible explanation for the increased sleepiness during the first night shift is the pronounced shift in diurnal timing, which affects sleep, activity, meals, light exposure, etc. In particular, extended wakefulness might play a role. Research suggests that the time awake since the last main sleep is longer before the first night shift than before subsequent ones (45), which may increase homeostatic sleep pressure and, thus, sleepiness substantially during the first shift. Furthermore, the apparent decrease in sleepiness during consecutive night shifts may reflect partial habituation or adaptation. The particularly high level of sleepiness during the first night shift could act as a subjective reference point, making subsequent nights feel less demanding, even if actual sleepiness levels remain the same.

As previously discussed regarding shift length, effort-related compensation processes may also play a role. Workers may counteract rising sleepiness by increasing their effort, the consequences of which only become apparent during subsequent extended rest periods, when such compensation is no longer required. Supporting this assumption, studies have found that sleepiness (40) and reduced alertness (46) are worse on the first rest day following night shifts than during the shifts themselves. Future research should investigate whether, and how, these potential compensation costs vary with shift number (and length).

Overall, our results show the following: (i) sleepiness is higher during night shifts than during day shifts and is lowest during afternoon shifts; (ii) sleepiness increases during night shifts (up to around the 7^th^ hour), but not during day shifts; and (iii) sleepiness is particularly high during the first night of a shift sequence. Taken together, these findings strongly support the influence of circadian and homeostatic factors on sleepiness but provide limited evidence for time-on-duty or work-related effects – except in relation to the length of the night shift, where circadian, homeostatic, and time-on-duty effects may interact.

As discussed, this limited evidence may partly reflect the ongoing debate about whether, unlike other forms of fatigue, sleepiness is primarily influenced by circadian and homeostatic factors rather than time on duty (8). If this is the case, measures of subjective sleepiness may be less effective at detecting the cumulative effects of sustained work demands and may therefore underestimate certain risks associated with work-related fatigue. As Phillips (47) observed, "sleepiness alone fails to explain all of the important performance effects related to tiredness." In line with this notion, some studies report divergent results between measures of sleepiness and performance (eg, KSS versus PVT; 48), despite assessing related constructs. These differences may be due to masking factors, such as contextual or motivational influences, which affect subjective and objective measures of fatigue-related concepts differently (12). This may also help to explain why our findings differ from those of the meta-analysis by Fischer et al (6), which found an increased risk of occupational injuries with longer shift duration and more consecutive day and night shifts. Besides, it highlights the need to extend meta-analytic efforts to additional fatigue-related constructs.

### Further limitations and future research directions

In addition to the conceptual limitation that sleepiness may not capture all fatigue-related risks associated with certain working hours, our study is further limited by its exclusive focus on sleepiness as measured by the KSS. Although the KSS is a well-validated scale (18), it relies on self-reported data. Therefore, we cannot rule out potential biases due to contextual or motivational masking factors (12), for instance, in work settings where alertness is expected. However, since fatigue is a broad concept (9), there is currently no universally accepted standard measure. All operationalizations, including the KSS, capture only specific aspects of fatigue.

Furthermore, despite conducting a broad literature search, the meta-analysis focused on only three specific work schedule characteristics. Other relevant factors, such as rest breaks and rest periods between shifts, were not included even though there is evidence showing that they affect sleepiness (eg 49,). While future reviews could address these aspects, our research revealed that primary studies investigating sleepiness in relation to rest breaks and periods, especially those measuring sleepiness during these time windows, are still rare. Thus, more primary research is needed first.

Moreover, some methodological limitations should be noted. Several analyses were based on a small number of available studies, increasing the risk of second-order sampling error (50). Additionally, substantial or high heterogeneity across studies was often observed, however, this was expected given the diversity of the included studies, which were mostly from various field settings. Sensitivity analyses generally supported the robustness of the results, and moderator analyses helped address the heterogeneity further. However, data on some potentially important confounding variables was often missing, limiting our ability to control for them. These variables include organizational aspects, such as workload and shift sequence (eg, specific rosters or days off prior to a given shift), and individual aspects, such as chronotype and family and household responsibilities. Therefore, future studies should investigate and report on these variables more consistently. Finally, our analyses only included peer-reviewed journal articles. Nevertheless, tests for publication bias indicated that potential biases should be considered but are unlikely to have fundamentally changed the overall pattern of results.

### Practical recommendations

Our results highlight that sleepiness is higher during night compared to day shifts, particularly during the later hours of the shift and the first night in a sequence. Accordingly, the key practical implication is that night shift schedules should be designed to specifically mitigate these elevated risks of sleepiness. For example, shortening shift duration and ensuring sufficient rest before and during the first night could be beneficial.

In addition to scheduling adjustments, other measures, such as changes to the nature and intensity of work are advisable, even though these were not the focus of this meta-analysis. If possible, both monotonous work (51) and high workload (52) should be avoided during times of increased risk of sleepiness, particularly during the first night shift, as these could further exacerbate it. Furthermore, providing facilities for napping, such as quiet rooms, may help reduce excessive sleepiness (53). However, napping can lead to sleep inertia upon awakening, a risk that must be considered and, when possible, mitigated.

Organizations may also consider offering training on sleepiness and broader sleep and fatigue management. For instance, one individual fatigue management strategy is to take a short nap before the first night shift to reduce the amount of time spent awake beforehand (54). However, failures in managing fatigue are often not due to a lack of knowledge but rather the difficulty in translating such knowledge into action (53). Therefore, interventions should focus on supporting workers' abilities to self-regulate their thoughts, emotions, and behaviors in relation to sleep and fatigue (53) and living conditions.

Taken together, these measures could help mitigate sleepiness-related risks in (night) shift work and thereby maintain work performance and, above all, protect workers' health and safety.
